# Suicide Prevention Strategies in Europe: A Comparative Analysis of National Approaches

**DOI:** 10.1192/j.eurpsy.2025.516

**Published:** 2025-08-26

**Authors:** F. S. A. M. Swilem, A. Brezina, A. Tarrada, D. Ori, S. M. Hein, A. Pouloutidou, K. Silagadze, M. Gebele, O. Giannakopoulos, M. Skrobo, M. Jeurissen, I. Vandeputte, E. I. Guliyev, G. F. Fucho-Rius, C. Alla, I. Jora-Boerosu

**Affiliations:** 1 Child and Adolescent Psychiatry, Cambridge and Peterborough NHS foundation Trust, Peterborough, United Kingdom; 2 Department of Neurology, Centre Hospitalier de Luxembourg, Luxembourg, Luxembourg; 3 Department of Neurology, University Medical Center of Nancy, France, France; 4 Institute of Behavioural Sciences, Semmelweis, Budapest, Hungary; 5 Child and Adolescent Psychiatry, North East London NHS Foundation Trust, London, United Kingdom; 6 Department of Psychiatry, Aristotle University pf Thessaloniki, Greece, Greece; 7 Department of Psychiatry, National family Medicine centre , Tbilisi, Georgia; 8 Subacute Psychiatry Department, Gintermuiza Hospital, Latvia, Latvia; 9 Department of Psychiatry, 251 HAF General Hospital, Athens, Greece; 10 Department of Psychiatry, UHC Sestre Milosrdnice, Zagreb, Croatia; 11 Department of Addiction, LVR-Clinic, Duren, Germany; 12 Department of Psychiatry, Onze Lieve Vrouw, Bruges, Belgium; 13 Department of Psychiatry, Istanbul Faculty of Medicine, Istanbul, Türkiye; 14 Department of Psychiatry and Legal Medicine, Institut d’Investigacio Parc Tauli (I3PT), Barcelona, Spain; 15 Medeferent-P Clinic, Chisinau, Moldova, Republic of; 16 The Mind Clinic, Bucharest, Romania

## Abstract

**Introduction:**

Inspired by discussions at the EPA Summer School 2024 on suicide prevention, this poster explores local strategies implemented across European nations. Emphasizing the importance of tailored approaches, the study analyses successful initiatives and community-based programs, and investigates how country-specific factors influence suicide rates. Key findings from research papers on innovative methodologies were also examined, offering insights to inform future practices in suicide prevention.

**Objectives:**

The poster aims to:

- Present local strategies for suicide prevention across European countries, focusing on the contributions of clinicians who attended the EPA Summer School.
- Highlight how knowledge of diverse strategies can impact clinical practice in mental health across Europe.

**Methods:**

- **Categorization:** European countries are grouped by suicide rates—high, medium, and low—using WHO and European CDC data.
- **Summarization:** Local prevention strategies and programs in each group are summarized.
- **Analysis:** Various socio-economic and cultural factors influencing suicide rates are discussed, including stigma, economic conditions, and access to healthcare.
- **Comparative Approach:** Strategies are compared to identify common successful elements and contextual challenges.

**Results:**

**Conclusions**

Local strategies tailored to national contexts are essential in suicide prevention efforts. Comparing diverse strategies reveals that approaches addressing cultural and economic factors hold the most promise for reducing suicide rates across Europe. This analysis underscores the need for continued cross-border collaboration and the exchange of best practices to create more effective, context-specific interventions.

**Disclosure of Interest:**

None Declared

